# Supplementary material for: Trends in breast cancer incidence in Ho Chi Minh City 1996–2015: A registry-based study
Source: PLoS One. 2021 Feb 10;16(2):e0246800. doi: 10.1371/journal.pone.0246800 (PMC7875422; doi:10.1371/journal.pone.0246800)
Supplement: Attachment — Submitted filename: Comments for Trends in breast cancer incidence....pdf [file pone.0246800.s001.pdf]

Comments for article "Trends in breast cancer incidence in Ho Chi Minh City 1996 - 2015: a registry-based study"

This article deals with the evolution of breast cancer incidence in Ho Chi Minh City of Vietnam country between 1996 to 2015. This is an important issue, since such analyses enable a measurement of the burden for the health system, to make choices for health policy.
Nevertheless, such analyses require three major things that also need to address in this article

1. How far are the data reliable? How can we be sure of the exhaustivity of the data, from 1996 to 2015 in specified country? The effects observed may be just an effect of a better registration of the cancer cases. A detailed description of methods of registration in that country should be provided, to discuss the evolution of incidence rates.
2. To discuss the observed trends, we need information about health facilities in underline country and health policy concerning breast cancer.
3. The application of relevant statistical methods are major concern to get the precise results.

Therefore, last two points are lacking and make this work difficult to appreciate, although the subject is of real importance and manuscript comprises of a large data set but needs major revision in terms of data analysis, technical writing, sentence structure improvement, and removal of grammatical errors.

Major highlights are pointed out as follow:

Introduction

Line 79-80: "Vietnam is the 15th most populous country in the world, with a population of 97 million (2020 statistics). Please Cite reference here.
Additionally, breast cancer risk factors should also be addressed in introduction.

Study design and method

This section needs to include relevant statistical methods used for data analysis. It is recommended that use median and IQR for nonparametric data (age diagnosis) and use kruskal wallis test to measure the difference among age groups of diagnosed cases in case of scale measurements and use chi-square where percentages are compared**.** Further, Join-point regression can be used for trend analysis.
Line 115-116 "we computed the point incidence of breast cancer for each 5-year period… 2010, and 2011 - 2015 inclusive". Briefly, mention how that incidence rate was calculated?

Line 118-119: “we calculated the age-standardized incidence rate for each of the 4 periods…” How age-standardized incidence rate was calculated?

Line 119-121: “We also employed a segmented Poisson regression model to estimate the change in the incidence of breast cancer over time. All statistical analyses were conducted using the R Statistical Environment….”.

It is recommended that use join-point regression technique and report your results in form of estimated annual percentage change (EAPC) with 95% UI. Please mention R version and package name that used for analysis.

Results

Line 125: findings (13,498 women, or 95%) and same in abstract, are inconsistent with results calculated in Table 1. (Table 1 has 13,948 women, 98%). Additionally report p-value of test difference and chi-square value.

Line 133-138, “The average at diagnosis was 52 years (SD 11.6) and 56.3 years (13.4) for women and men, respectively. In women, there was a slight but statistically significant increase …….(Table 2)”.

Firstly, data is nonparametric so authors should report median with IQR for age diagnosis, rather than mean and SD. Author reported median age in abstract but results section included mean age? Please be consistent. Authors reported that “in women statistically significant increase in the average age of diagnosis of breast cancer during 1996 and 2015….”. This difference look non-significant; please report p-value with test statistic value. Also, include these results in Table. Table 2 can be revised using joint-point regression estimate (EAPC with 95%UI for each duration).

Line:143-149: “Segmented regression indicated that there were two trends in the incidence of breast cancer in women: the first period occurred between ….(Figure 1). Further analysis showed that there was a statistically significant increase in the age-specific incidence of breast cancer over the period of 1996 and 2015, and the increase ….(Figure 2). In women, the increase in the age-specific incidence rate was observed among those aged…..”.

Join-point regression is widely used trend analysis technique and segmented regression is a part of it. Therefore, it is suggested that use main name of the technique for convenience of the readers.

Figure 1 and 2 , are not readable. A better presentation is needed here. Author should draw the trends across ages, years and cohort by year and age group (e.g. within age Group, within year and within cohort). Through these 3 figures Table 2 results can be well representative.

Line 160: Table 3. Briefly explain how the standardization was performed (ASR)?

Conclusion

Line 53-54: "These very first data from Vietnam suggest that although the incidence of breast cancer in Vietnam remains relatively low, it has increased over time, and that the increase was mainly attributable to those age groups of 50 and 70".

Finally, authors concluded that "Our data also confirm that Vietnamese women tend to have breast cancer at younger ages compared to Caucasian women". Younger ages? Conclusion is not consistent with the findings. (Further, most of the GBD studies reported that women breast cancer is more prevalent in older ages worldwide). Make any changes to the abstract that align with those made in the text.

Minor comments

Line 34: Revise sentence structure (In line with the related literature, for example, see following literature)

1. (Nguyen TP, Luu HN, Nguyen MV, Tran MT, Tuong TT, Tran CT, Boffetta P. Attributable Causes of Cancer in Vietnam. JCO global oncology. 2020 Feb;6:195-204.
2. Nguyen SM, Deppen S, Nguyen GH, Pham DX, Bui TD, Tran TV. Projecting cancer incidence for 2025 in the 2 largest populated cities in Vietnam. Cancer Control. 2019 Jul 22;26(1):1073274819865274.
3. Pham T, Bui L, Kim G, Hoang D, Tran T, Hoang M. Cancers in Vietnam—burden and control efforts: a narrative scoping review. Cancer Control. 2019 Jul 17;26(1):1073274819863802.)

Line 51: age-standardized incidence rate, replace it with ASR as its already mentioned in the abstract.

Line 55: those age groups of 50 and 70 years…

Line 60: 2019 showed that approx……..

Line 61: improper sentence structure.. What is meant by was also the most..?

Line 69: is lower than in Caucasian populations…….. remove "in"

Line 71 & 72. Mention reference 5 once after the completion of related information.

Line 73: women tends to have breast cancer in …….

Line 125: write as 1st Jan. 1996……

Line 127-128: Rewrite these lines

Line 133: word age is missing, modify as … The average age at diagnosis was 52 years ….

Line 145: later instead of latter

158: repetitive words in one sentence

Line 189: revise the sentence

198: unable to understand the statement

Line 202: Asian women tends to ….

Line 224: cancer incidence rates in Vietnam's urban population remain …..
