# Supplementary material for: Trends in breast cancer incidence in Ho Chi Minh City 1996–2015: A registry-based study
Source: PLoS One. 2021 Feb 10;16(2):e0246800. doi: 10.1371/journal.pone.0246800 (PMC7875422; doi:10.1371/journal.pone.0246800)
Supplement: Attachment — Submitted filename: Response to comments.docx [file pone.0246800.s004.docx]

**PONE-D-20-16460, Trends in breast cancer incidence in Ho Chi Minh City 1996 - 2015: a registry-based study**

**Response to Reviewers' Comments**

*"Reviewer #2: The article remain several spelling and gramma error. Some technical error mention in commend should be addressed (attached track-change version)."*

**Authors:** **Thank you very much for your help and the track changes. We have gone through the changes and corrected them. We however want to retain some technical terms (eg 'inflexion point', 'ljr', "individual person's data").**

"*Reviewer #3: The authors have improved the presentation of their work and answered most of my queries satisfactorily. However, I remain very concern about the following points. Therefore, before it can be published, the following points need to clarify in the manuscript.*

*1. Where authors use the word significant or statistical significant with the results reported, give the p-value or 95% uncertainty interval with that findings, e.g. line 50, 145."*

**Authors: As we stated previously, this study aimed at describing the trend of breast cancer incidence over the 1996-2015 period; it was not designed to test any hypothesis that requires P-value.**

*"2. For better presentation of results in table 2 it is suggest to authors, insert one column in Table 2 with p-value heading and report p value there for women and men separately, that will indicate the significant difference among periods by ages."*

**Authors: This table is purely descriptive. The table is not meant to test any hypothesis. We consider that the actual ASR data are much more important and much more informative than any P-value which is largely sample size dependent.**

*"3. To make article results more interesting for readers, I would like to suggest to authors, also draw estimated annual incidence rate by cancer type, e.g. compare Malignant and other cancer type trend (like figure 1), If authors have sufficient data for type of cancer by period."*

**Authors: We appreciate your suggestion. However, more than 99% of the cases were malignant; only 0.3% were classified as in-situ.**

"*4. Line 48, Authors reported, “The ASR of breast cancer during 2011-2015 period was 21.5 cases per 100,000 women…”. Why the ASR reported in line 48 and in Table 3 are inconsistent, e.g. women ASR during 2011-2015. Brief clarification needed on how these ASR was calculated? Also need to clarify or fix this confusion*."

**Authors: The number quoted in the Abstract is the ASR per year, whereas the number quoted in table 3 refers to the 5-year ASR (2011-2015 period). For consistency, we have used the 5-year rate in the Abstract.**
